# Assessing Child Feeding Practices Among Parents of Hospitalized Children and Their Associated Factors in a Tertiary Care Hospital in Bhubaneswar, India: A Descriptive Cross-Sectional Study

**DOI:** 10.7759/cureus.98420

**Published:** 2025-12-03

**Authors:** Sumana Samanta, Asha P Shetty

**Affiliations:** 1 Pediatrics, All India Institute of Medical Sciences, Bhubaneswar, Bhubaneswar, IND

**Keywords:** factors associated feeding practices, feeding challenges, hospitalized children, socio-demographic factors, parental feeding practices

## Abstract

Background

Parental feeding practices play a vital role in determining the nutritional status and well-being of children, especially during hospitalization. Various factors, including socio-demographic characteristics, hospital environment, and child-specific conditions, influence feeding practices. This study aimed to assess child feeding practices among parents of hospitalized children and identify the associated factors in a tertiary care hospital in Bhubaneswar, India.

Methods

A descriptive cross-sectional study was conducted among 380 parents of hospitalized children aged 12-36 months. Data were collected using a structured interview schedule and a feeding practices rating scale. The data were analyzed using IBM SPSS Statistics for Windows, Version 21 (Released 2012; IBM Corp., Armonk, New York, United States), applying descriptive and inferential statistics, including chi-square tests and logistic regression analysis.

Results

The study findings revealed that 219 (57.6%) of parents had unsatisfactory feeding practices, while 161 (42.4%) exhibited satisfactory feeding practices. Key factors associated with feeding practices included: physical limitations or difficulties with eating; the child’s appetite in the hospital; feeding challenges such as refusal, gagging, and vomiting; hospital environment; and feeding management during medical procedures and eating behavior. Additionally, a significant association was found between feeding practices and the current weight of the child and socioeconomic status. Logistic regression analysis revealed that three significant factors were associated with feeding practices among hospitalized children: the children’s current weight (odds ratio (OR) = 2.935, P < 0.001), socioeconomic condition (OR = 2.476, P = 0.009), and feeding management during medical procedure (OR = 0.135, P < 0.001).

Conclusion

Based on the present study, the following conclusions can be made that more caregivers had unsatisfactory feeding practices because the children had various kinds of illness, an unfamiliar hospital environment, physical limitations, and difficulties with eating. Moreover, a significant association was found between the child’s weight, socioeconomic conditions, and feeding management during the medical procedure with feeding practices.

## Introduction

Adequate nutrition during early childhood plays a vital role in ensuring optimal physical and cognitive development. Adhering to recommended feeding practices, including breastfeeding and appropriate complementary feeding, supports a child's overall well-being [1,2].

According to the World Health Organization (WHO) guidelines, infants should be exclusively breastfed for the first six months, after which complementary foods should be introduced while maintaining breastfeeding until at least two years of age [1]. Despite the recommendation of the WHO, inadequate infant feeding practices, combined with high rates of infectious diseases during the first two years of life, are the leading contributing factors to malnutrition [2]. Hospital-based evidence on feeding practices and malnutrition among Indian children remains limited; most available studies are community-based.

Improper complementary feeding increases the risk of undernutrition, illness, and mortality in infants and children under two years old. It is estimated that improving complementary feeding alone could prevent 6% of deaths in children under five [3]. There is a lack of regional hospital-based data related to feeding and hospitalization. Furthermore, research has shown that parents play a vital role in shaping their children's eating habits and weight development through their own eating behaviors and feeding practices [4].

Parental feeding practices are the methods parents use to guide and regulate their child’s eating habits during meals, and can significantly shape dietary behaviors and nutritional outcomes. These practices are modifiable and can alter children’s dietary habits [5]. They are shaped by a complex interplay of factors, including socioeconomic elements like family income and education, cultural background, personality traits, and psychological health. Furthermore, parents often adjust their feeding practices based on their child’s temperament, weight, eating habits, and their own perceptions and beliefs about these factors [6]. Thoroughly exploring the parental feeding practices and their associated factors can inform targeted and impactful interventions [7,8].

Feeding young children effectively requires parents or caregivers to possess adequate nutritional knowledge to ensure appropriate feeding practices, food choices, and calorie intake. Consequently, educational programs should be made available to parents of children of all ages (from infancy to adolescence) across diverse socioeconomic backgrounds, with an emphasis on promoting physical activity, limiting screen time (television, video games, and computers), and encouraging sufficient sleep. Parents should receive guidance on fostering long-term healthy habits, creating positive eating behaviors in their children, and identifying behavioral factors that contribute to malnutrition and eating disorders [9].

In India, the Comprehensive National Nutrition Survey (CNNS, 2016-2018) revealed several significant findings about feeding practices. Regarding breastfeeding initiation, they found that 57% of children were breastfed within the first hour of birth, with 83% of children still breastfeeding at 12-15 months, and for exclusive breastfeeding (EBF), they found that around 58% of infants under six months were exclusively breastfed. The study found that 53% of infants started complementary feeding at six months, but only 42% of children between six and 23 months had the minimum meal frequency, 21% had a diverse diet, and only 6% met the criteria for a minimum acceptable diet [10].

Hospitalized children are more vulnerable to high morbidity and mortality than the general population. During or after illness, children should be encouraged to eat soft, varied, and appealing foods, with increased fluid intake, including frequent breastfeeding, to support nutrition and recovery. Despite reduced appetite, continued complementary feeding is crucial to preserve nutrients, aid recovery, and promote catch-up growth. Extra food is needed until the child regains any lost weight and resumes healthy growth [11]. In rural areas, a study involving 271 informal caregivers in India found that 77.85% of caregivers did not alter breastfeeding practices during illness. A study conducted in Ethiopia involving 602 parents revealed that only 45% of parents followed recommended feeding practices for sick children. In Tembaro woreda, 21.3% of children were fed more frequently when ill, and factors like paternal education, maternal antenatal care visits, and prior exposure to information about sick child feeding were identified as predictors for better feeding practices [12]. Despite these positive findings, many caregivers still face challenges when it comes to feeding sick children adequately. A study in South Asia highlights that infant and young child feeding (IYCF) during and after common childhood illnesses remains far from optimal. While most children (up to 98%) continue breastfeeding during illness, a significant number (up to 49%) are breastfed less frequently than usual, which is contrary to recommendations to increase breastfeeding during illness to meet the child’s higher nutritional and fluid needs. Furthermore, up to 75% of children have their complementary foods restricted in terms of frequency, quantity, and quality due to factors such as anorexia, caregivers' lack of awareness, traditional beliefs, and inadequate counseling from health workers. Health providers rarely advise mothers to increase breastfeeding frequency or encourage feeding soft, varied, and preferred foods during illness, which further exacerbates the issue [13]. A qualitative study from South Kivu identified poverty, lack of counseling, cultural food restrictions, and reduced appetite during illness as major barriers to optimal feeding [14].

Feeding practices in children are shaped by various factors, such as socioeconomic status (SES), maternal education, and health conditions. Several studies have explored these factors in different contexts, shedding light on how they shape feeding behaviors and practices, especially in the context of hospitalization and childhood illness [1,14].

Studies have shown that the educational level of mothers significantly impacts feeding practices. Mothers with higher education levels are more likely to engage in optimal feeding practices, especially concerning the timing of complementary feeding [15,16]. A hospital-based study conducted in Eastern India assessed the determinants of feeding behavior of children aged one to three years, with a particular focus on factors like feeding problems and the role of the mother. The study revealed that children who were fed by caregivers other than their mothers were more likely to experience undetected feeding problems. Furthermore, children born preterm were universally found to have eating difficulties. This suggests that maternal involvement in feeding is crucial, particularly in the hospital setting, where children may already be vulnerable due to their health condition [17].

While numerous studies exist on the feeding practices of children in community areas, there is a noticeable gap in research specifically addressing hospitalized children; hence, this study will enlighten the feeding practices of parents of hospitalized children.

This study seeks to examine parental feeding practices and the factors associated with them among hospitalized children. The objectives for the study include: (1) to assess the child feeding practices of parents of hospitalized children, (2) to identify the factors associated with feeding practices among hospitalized children, and (3) to determine the association between feeding practices of parents and sociodemographic variables.

## Materials and methods

A descriptive cross-sectional study was conducted to assess the feeding practices of parents and associated factors among hospitalized children aged 12-36 months. The study was carried out in the pediatric wards of the All India Institute of Medical Sciences (AIIMS), Bhubaneswar, India, a tertiary care hospital serving eastern India.

The study population consisted of parents of children aged 12-36 months who were admitted to pediatric medical, surgical, and hemato-oncology wards for a minimum of three days. A total enumerative sampling technique was used. The required sample size was calculated using the formula for cross-sectional studies, based on a previous prevalence (p = 45%) of feeding practice during illness [11]. At a 95% confidence level and 5% margin of error, the formula was used: $n = \frac{Z^2 p q}{d^2}$, where p = prevalence, q = (1-p), and d = absolute precision.

By substituting Z = 1.96, p = 45, q = 55, d = 5%,

$n = \frac{(1.96)^2 \times 45 \times 55}{5^2}$

$n = \frac{1.96 \times 1.96 \times 45 \times 55}{25}$

$n = \frac{9507.96}{25}$

n = 380 samples

The final sample size was 380. The inclusion criteria were as follows: parents of children aged 12-36 months admitted for at least three days; children who were receiving oral feeding; and parents who were available and willing to participate during data collection. The exclusion criteria were as follows: children on nil per os (NPO) status; children receiving enteral or parenteral feeding; and parents who either declined participation or had significant language barriers.

Three tools were used for data collection: a structured interview schedule divided into two sections - Section A: Child’s demographic details (age, gender, weight, birth order, hospitalization history, dietary restrictions) and Section B: Parental demographics (age, education, occupation, SES, type of family, residence) (see Appendix A).

Another tool used to assess feeding practices, developed by the researcher, consists of 24 items across five domains: nourishment, feeding style, food hygiene, food refusal, and feeding approach, scored on a 5-point rating scale (Always = 4 to Never = 0); Total score range: 0-96. Based on the median value (M = 65), the scoring was divided into two categories: Unsatisfactory (0-65) and Satisfactory (66-96) (see Appendix B).

A structured interview schedule was used to assess the associated factors, which included five domains: physical, social, psychological, environmental, and interpersonal. The schedule consisted of closed-ended questions identifying factors influencing feeding practices. Content validity was reviewed by seven experts from pediatrics and nursing faculties; the content validity index (CVI) was 0.9 for the feeding practice scale and structured interview schedule to assess factors associated with feeding practices. The experts who reviewed the content validity of the tool had no conflict of interest with the authors or with the publication of this article. The tools were translated into Odia and Bengali, followed by back-translation, and reliability testing was performed using Cronbach’s alpha for the feeding practices scale, yielding an r-value of 0.812 (see Appendix C).

Ethical clearance was obtained from the Institutional Ethical Committee, AIIMS Bhubaneswar (Ref. No.: IEC/AIIMS BBSR/Nursing/2024-25/17). Informed consent was obtained from all participants, and confidentiality and anonymity were maintained. Data collection was conducted solely by the author. Before the main data collection, a small-scale pilot study was performed to assess interviewer consistency. Although the interviewer was not formally trained, consistent procedures were maintained throughout the study, and data were collected carefully. To minimize bias, the author ensured a neutral tone, appropriate body language, avoidance of leading questions, and maintenance of participant privacy. Data were collected between 11 November and 24 December 2024. Eligible participants were approached in the pediatric wards. After obtaining consent, demographic data were collected through interviews, and participants self-rated their feeding practices using the translated scale. Data on associated factors were also collected through interviews.

Data were coded and analyzed using IBM SPSS Statistics for Windows, Version 21 (Released 2012; IBM Corp., Armonk, New York, United States). Descriptive statistics, such as frequency, percentage, median, and interquartile range, were calculated, and inferential statistics were calculated by using the chi-square test, Fisher’s exact test, and binary logistic regression.

## Results

As shown in Table 1, the majority were aged 12-20 months, 160 (42.1%), and 235 (61.8%) had normal weight as per WHO growth standards, 243 (63.9%) had birth weight >2500 grams. Child characteristics also indicated that 255 (67.1%) were male, 213 (56.1%) had prior hospitalization, and among them, 36 (16.9%) had dietary restrictions.

**Table 1:** Frequency and Percentage Distribution of Sociodemographic Variables of Children (n = 380)

Sl. no.	Variables	Frequency (f)	Percentage (%)
1	Age of child (in completed months)		
	12-20	160	42.1
	21-29	115	30.3
	30-36	105	27.6
2	Birth order of the child		
	First order	168	44.2
	Second order	191	50.3
	Third order	15	3.9
	Above 3	6	1.6
3	Birth weight of child (in grams)		
	<2500	137	36.1
	>2500	243	63.9
4	Current weight of the child (as per WHO)		
	Normal weight	235	61.8
	Underweight	133	35
	Overweight	12	3.2

Parental characteristics include that most caregivers were aged 21-25 years, 214 (56.3%), 157 (41.3%) of household heads had primary education, 177 (46.6%) belonged to the elementary occupational group, 263 (69.2%) families were from the upper-lower SEC (as per modified Kuppuswamy scale), 319 (83.9%) belonged to joint families, and 332 (87.4%) resided in rural areas.

**Figure 1:**
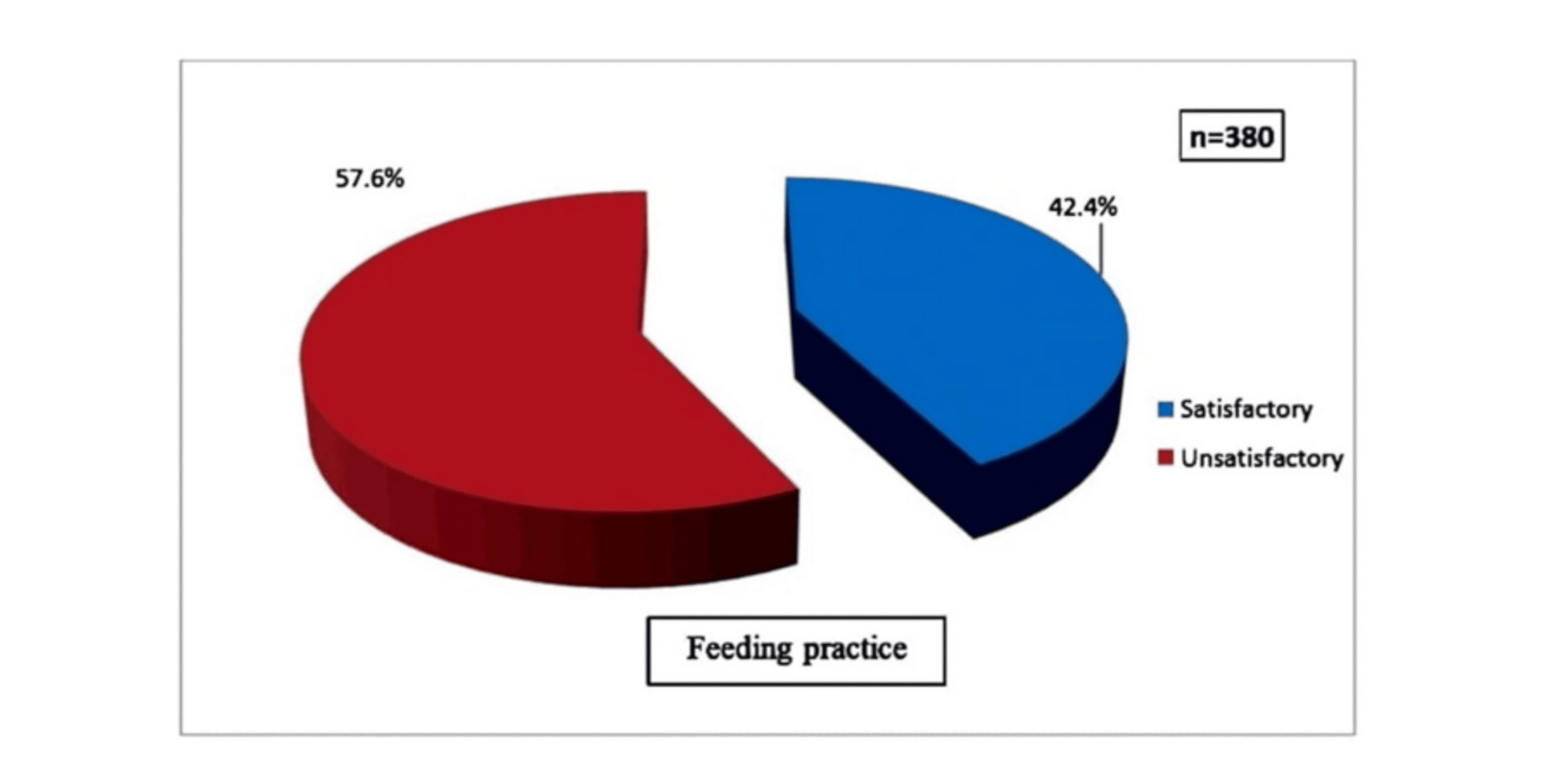
Feeding Practices Among Parents of Hospitalized Children

As shown in Figure 1, 219 (57.6%) of parents had unsatisfactory feeding practices, and 161 (42.4%) had satisfactory feeding practices.

The domain-wise analysis of feeding practices shown in Table 2 shows that the highest median score was recorded for the feeding style by caregiver at the hospital (median = 17), and the lowest median score was for food refusal (median = 5) in the feeding practice scale.

**Table 2:** Domain-Wise Scores for Feeding Practice (n = 380)

Sl. no.	Domains in the feeding practice scale	Minimum score	Maximum score	Median (IQR)
1.	Nourishment at the hospital (four items)	4	16	15 (9)
2.	Feeding style by caregiver at the hospital (six items)	6	24	17 (3)
3.	Food hygiene at the hospital (five items)	5	20	16 (3)
4.	Food refusal by a child in the hospital (three items)	3	12	5 (3)
5.	Feeding approach by caregiver at the hospital (six items)	6	24	13 (3)

The factors associated with feeding practices includes: Physical difficulties in eating (χ^2^ = 7.79, P = 0.005), poor appetite in hospital (χ^2^ = 10.38, P = 0.01), feeding challenges like gagging/refusal (χ^2^ = 19.61, P < 0.001), hospital environment (χ^2^ = 8.74, P = 0.03), feeding during procedures (χ^2^ = 18.15, P < 0.001), SES (χ^2^ = 11.69, P = 0.006) and child’s current weight (χ^2^ = 26.49, P < 0.001).

As shown in Table 3, to identify predictors of satisfactory feeding practices, binary logistic regression was performed. Significant predictors included: current weight of the child (odds ratio (OR) = 2.935, P < 0.001), SES (OR = 2.476, P = 0.009), and feeding management during medical procedures (OR = 0.135, P < 0.001).

**Table 3:** Binary Logistic Regression Results for Feeding Practices and Associated Factors (n = 380) * P-value < 0.05 is significant; CI: confidence interval

Sl. no.	Predictor variable	Odds ratio (OR)	95% CI	Chi-square		Sig. (P-value)
1	Current weight of the child (as per WHO) - Normal weight/Abnormal weight (Reference)	2.935	1.855-4.644		4.644	0.000*
2	Socio-economic status - Upper/Lower (Reference)	2.476	1.251-4.897		6.783	0.009*
3	Physical limitations or difficulties with eating - Yes/No (Reference)	0.722	0.441-1.181		1.684	0.194
4	Child's appetite in the hospital - Good Poor (Reference)	1.396	0.839-2.324		1.648	0.199
5	Feeding difficulties or challenges in the hospital - Yes/No (Reference)	0.970	0.424-2.219		0.005	0.942
6	Hospital environment influences child’s eating - Yes/No (Reference)	0.418	0.167-1.045		3.479	0.062
7	Feeding management during medical procedure/treatments - Yes/No (Reference)	0.135	0.048-0.381		14.313	0.000*
8	Feeding practice influences eating behavior - Yes/No (Reference)	0.865	0.537-1.393		0.355	0.551

## Discussion

The present study revealed that 160 (42.1%) of the hospitalized children were in the age group of 12-20 months, which was congruent with the finding of a study conducted by Fanta and Cherie, where 45.8% of the children were age group between 12 and 17 months [18]. A similar finding was reported in a study conducted by Sağlam et al., which is also in support of the present study findings, which reported that early childhood (12-24 months) is a critical stage for dietary transitions and growth, making feeding practices particularly important during this period [1].

The present study also revealed that 255 (67.1%) of the hospitalized children were male and 125 (32.9%) were female, which was congruent with the findings of a study conducted by Sağlam et al., where 51% were male, and 49% were female [1].

Regarding birth order, the study found that 191 (50.3%) of children were second-born, while only six (1.6%) were third-born or above. This indicates that a high proportion of families in our sample had more than one child. A similar study finding by Fanta and Cherie indicated that 63.1% were third and above order, which also indicated a high proportion of multi-child families [18].

Regarding birth weight, the present study found that 243 (63.9%) of children had a birth weight greater than 2500 grams, showing a relatively healthy birth-weight distribution. These findings were comparable with the study conducted by Ganesan et al., where 75.78% children had a normal birth weight (≥2.5 kg) [19].

In terms of nutritional status, 133 (35%) of children in the present study were underweight, while 12 (3.2%) were overweight. Comparing with the present study, Ganesan et al. reported slightly higher malnourished children (42.3%) in an urban-based tertiary care hospital, South India [19]. The current study also revealed that 235 (61.8%) of children were of normal weight, which is slightly higher than the findings of Ganesan et al., who reported a lower proportion of children maintaining normal weight (46.38%) in a hospital setting [19].

The present study also revealed that 319 (83.9%) of the study samples were from a joint family, which was congruent with the study conducted in South India, indicating that 50.53% respondents were from a joint family [19]. The present study also presented that 263 (69.2%) of the study participants belonged to upper and lower socioeconomic conditions as per the modified Kuppuswamy scale (2023).

Feeding practices were assessed using a feeding practice scale, and the results categorized parents as having either satisfactory or unsatisfactory feeding practices. The study found that 219 (57.6%) of parents had unsatisfactory feeding practices, while 161 (42.4%) demonstrated satisfactory feeding practices. This was congruent with the study finding, which was conducted by Hailu et al., indicating that only 45% of parents followed recommended feeding practices [11]. Similar study findings were reported by Kasse et al., who found that 54.4% of mothers demonstrated good feeding practices for their sick children [20].

Various factors were found to be associated with feeding practices, including the child’s weight, SES, and environmental influences. A significant association was observed between feeding practices and the child’s weight (ꭓ^2 ^= 26.49, P < 0.001), with underweight children more likely to experience unsatisfactory feeding. This finding aligns with research conducted by Mekuria, who found that children with lower weight-for-age often had inadequate dietary intake [12].

SES was also a key determinant of feeding practices. The study found a significant association (ꭓ^2^ = 11.69, P = 0.006) between feeding practices and family income. Similar findings were reported by Ganesan et al., who emphasized that financial stability allows families to ensure better nutritional quality for their children [19].

Hospital-related factors also influenced feeding practices. The present study found that physical limitation, the child’s appetite, difficulties with eating, distractions, and unfamiliar surroundings significantly impacted the child. This finding is in agreement with research conducted by Hailu et al., which highlighted that hospitalized children often exhibit reduced appetite due to environmental stressors [11]. Additionally, feeding management during medical procedures was significantly associated with feeding practices (ꭓ^2^ = 18.15, P < 0.001), reinforcing the importance of structured mealtime routines in hospital settings. Hence, the null hypothesis was rejected, and the research hypothesis was accepted, and it is inferred that the feeding practice was associated with demographic data, including the current weight of the child and the socioeconomic conditions of the child.

There was no significant association found between the current medical conditions of the child and dietary restrictions, and also there was no significant finding between feeding practice and counseling received from IYCF, which was quite similar to the study findings conducted by Sağlam et al. [1].

The following were the limitations of the study: (a) Since the study relies on parental responses, there may be inaccuracies in recalling feeding practices; (b) the data on feeding practices were based on parental self-reporting, which may introduce social desirability bias; and (c) the study focused orally fed children so the generalizability is limited for all hospitalized children who are critically ill.

## Conclusions

Parental feeding practices are influenced by multiple factors; targeted support during hospitalization is essential. This study highlights the significant gaps in feeding practices among parents of hospitalized children aged 12-36 months in a tertiary care hospital. A majority of parents demonstrated unsatisfactory feeding practices, influenced by a variety of physical, social, psychological, and institutional factors. The present study has implications for nursing service, nursing education, nursing research, and nursing administration. Nurses should provide targeted counseling to parents regarding appropriate feeding practices based on the child’s age, illness condition, and nutritional needs. Incorporating parental feeding practices and nutritional guidelines into the nursing curriculum will help future nurses provide better guidance to caregivers. Future research should include enterally and parenterally fed children to assess feeding challenges across all hospitalized children. Observational studies can be done to get more accurate data regarding feeding practice, because the researcher personally felt that parents were answering correctly, while they were practicing the wrong things.

## Appendices

Appendix A

Structured interview schedule on sociodemographic variables of parents and the child.

Purpose - A structured interview schedule is developed to collect background data of study participants.

Instructions: The interviewer will tick (√) the right answer in the respective space according to response of the respondent or write in the space provided.

Section - A

Socio-demographic data of child: The investigator will fill this space; these data will not be included for analysis.

Clinical data of child: Date and time of data collection:

Date of admission:

I.P. number: Ward name:

Reason for admission:

Contact number:

1. Age (in completed months) -

2. Gender - 

a) Male

b) Female

c) Other

3. Birth order of child-

a) 1st

b) 2nd

c) 3rd

d) Above 3

4. Birth weight of the child (in grams) - 

5. Current weight of the child (as per WHO) -

a) Normal Weight

b) Underweight

c) Overweight

6. Previous hospitalization -

a) Yes 

b) No

7. If yes, Dietary restrictions due to previous hospitalization -

a) Yes (specify)

b) No

Section B

Socio-demographic data of parents

1. Age of mother/caregiver (in year) -

a) <20

b) 21-25

c) 26-30

d) >30

2. Educational qualification of head of the family (modified K.S. 2023) -

a) No formal education

b) Primary

c) Middle school

d) High school

e) Intermediate/diploma

f) Graduate

g) Professional degree

3. Occupation of head of the family (modified K.S. 2023) -

a) Legislators, senior officials, manager

b) Professional

c) Technicians/associate professional

d) Clerk

e) Skilled worker, shop and market sales worker

f) Skilled agricultural and fishery worker

g) Craft and related trade worker

h) Plant and machine operator and assemblers

i) Elementary occupation

j) Unemployed

4. Type of family -

a) Nuclear

b) Joint

c) Extended

5. Residential area -

a) Rural

b) Urban

6. Monthly income of family (in Rs) (modified K.S. 2023) - 

a) >146,104

b) 109,580-146,103

c) 73,054-109,579

d) 68,455-73,053

e) 63,854-68,454

f) 59,252-63,853

g) 54,651-59,251

h) 45,589-54,650

i) 36,527-45,588

j) 21,914-36,526

k) 7,316-21,913

l) <7,315

7. Socio-economic status (modified K.S 2023)-

 a) Upper

 b) Upper middle

 c) Lower middle

 d) Upper lower

 e) Lower

Appendix B

Feeding practice scale to assess feeding practices of parents of hospitalized children between 12 and 36 months.

Instructions: These questions deal with your interactions with your child when you are feeding the food to your child in the hospital. Kindly read the questions given below and answer them questions. Tick (√) the appropriate option according to you against each statement. The questions are divided into five sections. Provide one response per statement. Answer each statement properly that describes how often these interactions happen. There are five domains - Nourishment at hospital, Feeding style by caregiver at hospital, Food hygiene at hospital, Food refusal by child in hospital, Feeding approach by caregiver at hospital, respectively.

**Table 4:** Feeding practice scale *: Reverse scoring

Sl. no	Items	Always	Often	Sometimes	Rarely	Never
1.	My child is taking breastfeeding + commercial/ home based/ combination at hospital	4	3	2	1	0
2.	My child is taking only commercial/home based/combination feeding at hospital	4	3	2	1	0
3.	My child is taking family pot meal continue with breastfeed at hospital	4	3	2	1	0
4.	I always ensure my child gets enough fluids during hospitalization	4	3	2	1	0

Sl no	Items	Always	Often	Sometimes	Rarely	Never
1.	I feed my child more than four times in a day in hospital	4	3	2	1	0
2.	My child eats at set times in hospital	4	3	2	1	0
3.	I do control amount of feeding as per child’s appetite in hospital	4	3	2	1	0
4.	I force my child to eat or swallow food against their will while in the hospital*	0	1	2	3	4
5.	I allow my child to use mobile while feeding in the hospital*	0	1	2	3	4
6.	I make sure that my child is not lying or in comfortable position (e.g. sitting)while feeding in hospital	4	3	2	1	0

Sl no	Items	Always	Often	Sometimes	Rarely	Never
1.	I wash my hands with water and soap before preparing food in the hospital	4	3	2	1	0
2.	I make sure the children’s hands are washed with water and soap before they eat.	4	3	2	1	0
3.	I make sure that the plates, cups, spoons, and forks that are to be used are clean.	4	3	2	1	0
4.	I make sure the plates and cups that are to be used are not cracked or scratched.	4	3	2	1	0
5.	I make sure that the cooked food kept at room temperature is given to the children within 2 hours.	4	3	2	1	0

Sl no	Items	Always	Often	Sometimes	Rarely	Never
1.	When my child rejects food I encourage trying again without pressure	4	3	2	1	0
2.	When my child rejects food I offer alternative food immediately	4	3	2	1	0
3.	When my child rejects food I respect refusal without further offering	4	3	2	1	0

Sl no	Items	Always	Often	Sometimes	Rarely	Never
1.	I try to eat healthy foods always in front of my child in the hospital	4	3	2	1	0
2.	I encourage my child to finish all the food in front of him/her in the hospital	4	3	2	1	0
3.	I praise my child after each bit to encourage finishing the food in the hospital	4	3	2	1	0
4.	When my child refuses food in the hospital, they usually eat I encourage them by offering non-food reward (e.g. toy, etc.)	4	3	2	1	0
5.	When my child refuses food in the hospital, they usually eat I encourage them by offering food reward (e.g. Chocolates, etc.)	4	3	2	1	0
6.	I provide food in different shapes and sizes and serve the food in child’s favourite plate in the hospital	4	3	2	1	0

Appendix C

Structured interview schedule to assess factors associated with feeding practice.

Purpose - A structured interview schedule is developed to collect data regarding factors associated with feeding practices of parents of hospitalized children

Instructions: The interviewer will tick (√) the right answer in the respective space according to response of the respondent or write in the space provided.

Physical Domain

1. What is your child's current medical condition?

a) Acute illness (e.g., pneumonia, gastroenteritis)

b) Chronic condition (e.g., diabetes, cystic fibrosis)

c) Surgical recovery

d) Other (please specify)______________________

2. Are there any physical limitations or difficulties with eating?

a) Yes

d) No

3. How would you describe your child's appetite in the hospital?

a) Very poor

b) Poor

c) Average

d) Good

4. Has your child experienced any feeding difficulties or challenges in the hospital?

a) Yes, refusal

b) Yes, gagging

c) Yes, vomiting

d) Other (please specify)______________________

d) No

5. Are there any dietary restrictions or modifications needed for your child's health in the hospital?

a) Yes (please specify)_____________________

c) No

6. Does your child have any food dislikes or allergies?

a) Yes (please specify): ______________________

b) No

7. How often do you clean your child’s teeth during hospitalization?

a) Once a day

b) Two times a day

c) More than two times a day

d) No cleaning of teeth

Social domain:

1. Are there specific cultural practices during meals?-

a) Yes (specify)

b) No

2. Who typically interacts with your child during mealtimes in the hospital?

a) Parent/caregiver

b) Nurse

c) Other hospital staff (please specify)______________________

d) None

3. Have you received any counselling regarding Infant young child feeding?-

a) Yes (if yes specify where)______________________

b) No

Psychological domain:

1. Does your child have preference for the temperature of their food?-

a) Yes, if yes,

3. a.1 Hot

3. a.2 cold

3. a.3 Other (specify)

b) No

2. Does the visual presentation of food influence your child’s willingness to eat?

a) Yes

b) No

3. How does your child express their emotions related to food in the hospital?

a) Tantrums

b) Eagerness

c) Crying

d) Other (please specify)

4. Are there any psychological factors that affect your child's eating behavior in the hospital?

a) Separation anxiety

b) Fear of medical equipment

c) Other (please specify)

d) None

5. How would you describe your child's relationship with eating in the hospital?

a) Enjoyable

b) Stressful

c) Other (please specify)

Environmental Domain

1. Does your child have specific preferences in hospital regarding where they eat at home (e.g. high chair, family table)?-

 a) Yes

 b) No

2. How do you manage mealtime distractions (e.g., mobile phone, toys)?

a) Limiting distractions

b) Creating mealtime rules

c) Encouraging conversations

d) Other (please specify) ______________________

e) None

3. How do you think the hospital environment affects your child's eating behavior?

a) Distractions (e.g., noise, lights)

b) Unfamiliar surroundings

c) Limited food choices

d) Other (please specify)______________________

e) None

4. How do you manage feeding during medical procedures or treatments?

a) Timing meals around procedures

b) Providing comfort before/after

c) Other (please specify)______________________

d) None

5. How has your child's feeding routine changed since being hospitalized?

a) More frequent feedings

b) Less frequent feedings

c) Same as before being admitted to the hospital

d) Other (please specify):______________________

Interpersonal Domain

1. How do your feeding practices affect your child’s eating behavior?

a) Encourage healthy eating

b) Create mealtime routines

c) Allow flexibility in food choices

d) Other (please specify)______________________

e) None

2. How do you adapt your child’s diet to their specific health needs?

a) Follow specific diet

b) Provide nutrient rich food

c) Other (please specify)______________________

3. How do you ensure your child receives adequate nutrition in the hospital?

a) Advocating for specific foods

b) Monitoring intake

c) Other (please specify)______________________

4. What are your perceptions of your child’s weight?

a) Underweight

b) Overweight

c) Normal weight

d) Other (specify)______________________

5. How would you rate the support and guidance provided by the healthcare team regarding your child’s eating behavior?

a) Excellent

b) Good

c) Fair

d) Poor

e) Very poor

6. What challenges have you encountered regarding feeding your child in the hospital?

a) Lack of privacy

b) Limited food choices

c) Difficulty feeding in unfamiliar environment

d) Other (please specify)______________________

7. How do you collaborate with hospital staff regarding your child’s eating habits?

a) Providing information

b) Following staff recommendations

c) Other (please specify)______________________

## References

[1] Sağlam NÖ, Bülbül L, Kazancı SY, Hatipoğlu SS (2019). Factors affecting breastfeeding and complementary feeding choices for children aged 24 to 48 months. Sisli Etfal Hastan Tip Bul.

[2] Degefa N, Tadesse H, Aga F, Yeheyis T (2019). Sick child feeding practice and associated factors among mothers of children less than 24 months old, in Burayu town, Ethiopia. Int J Pediatr.

[3] Joshi N, Agho KE, Dibley MJ, Senarath U, Tiwari K (2012). Determinants of inappropriate complementary feeding practices in young children in Nepal: secondary data analysis of Demographic and Health Survey 2006. Matern Child Nutr.

[4] Jansen PW, Roza SJ, Jaddoe VW (2012). Children's eating behavior, feeding practices of parents and weight problems in early childhood: results from the population-based Generation R Study. Int J Behav Nutr Phys Act.

[5] Sandvik P, Kuronen S, Reijs Richards H, Eli K, Ek A, Somaraki M, Nowicka P (2022). Associations of preschoolers' dietary patterns with eating behaviors and parental feeding practices at a 12-month follow-up of obesity treatment. Appetite.

[6] Areja A, Yohannes D, Yohannis M (2017). Determinants of appropriate complementary feeding practice among mothers having children 6-23 months of age in rural Damot sore district, Southern Ethiopia; a community based cross sectional study. BMC Nutr.

[7] Costa A, Oliveira A (2023). Parental feeding practices and children’s eating behaviours: an overview of their complex relationship. Healthcare (Basel).

[8] Senarath U, Agho KE, Akram DE (2012). Comparisons of complementary feeding indicators and associated factors in children aged 6-23 months across five South Asian countries. Matern Child Nutr.

[9] Scaglioni S, De Cosmi V, Ciappolino V, Parazzini F, Brambilla P, Agostoni C (2018). Factors influencing children’s eating behaviours. Nutrients.

[10] Sharma M, Gaidhane A, Choudhari SG (2024). A review of infant and young child feeding practices and their challenges in India. Cureus.

[11] Hailu FM, Kefene SW, Sorrie MB, Mekuria MS, Guyo TG (2023). Sick child's feeding practices and associated factors among mothers with sick children aged less than 2 years in Gamo zone, southern Ethiopia. Does the participation of fathers contribute to improving nutrition? A facility-based cross-sectional study. Front Public Health.

[12] Mekuria T, Hassen K, Gali N (2020). Sick Child Feeding Practice and Associated Factors Among Mothers of Under Two Year Sick Children in Tembaro Woreda, Southern Ethiopia [Thesis]. Jimma Ethiopia: Jimma University.

[13] Paintal K, Aguayo VM (2016). Feeding practices for infants and young children during and after common illness. Evidence from South Asia. Matern Child Nutr.

[14] Zhu H, Zhao K, Huang L (2024). Individual, family and social-related factors of eating behavior among Chinese children with overweight or obesity from the perspective of family system. Front Pediatr.

[15] Varghese A, Agarwal M, Singh VK (2023). Complementary feeding practices in children aged 6-23 months in rural Lucknow: a cross-sectional study. Clin Epidemiol Glob Health.

[16] Kurnia ID, Rachmawati PD, Arief YS, Krisnana I, Rithpho P, Arifin H (2024). Factors associated with infant and young child feeding practices in children aged 6-23 months in Indonesia: a nationwide study. J Pediatr Nurs.

[17] Bhairavabhatla KC, Epari V, Panigrahi S (2020). Feeding behaviour and its determinants among healthy toddlers in an urban city of India. medRxiv.

[18] Fanta M, Cherie HA (2020). Magnitude and determinants of appropriate complementary feeding practice among mothers of children age 6-23 months in Western Ethiopia. PLoS One.

[19] Ganesan S, Jayaraj J, Geminiganesan S, Rajan M (2021). A study on parental awareness of feeding practices in children in the age-group 12-24 months. J Prev Med Hyg.

[20] Kasse T, Woldesilassie TS, Jisso AG, Lonsako AA, Haile A, Dejene YA (2024). Maternal feeding practices for sick children under 2 years in Wolkite town, Gurage Zone, Central Ethiopia, 2024: a community-based cross-sectional study. J Health Popul Nutr.

